# Experiences with and needs for aftercare following the death of a loved one in the ICU: a mixed-methods study among bereaved relatives

**DOI:** 10.1186/s12904-024-01396-5

**Published:** 2024-03-04

**Authors:** Sophie C. Renckens, Bregje D. Onwuteaka-Philipsen, Zina Jorna, Hanna T. Klop, Chantal du Perron, Lia van Zuylen, Monique A.H. Steegers, Birkitt L. ten Tusscher, Margo M.C. van Mol, Wouter de Ruijter, H. Roeline Pasman

**Affiliations:** 1 Department of Public and Occupational Health, Amsterdam UMC, location VU Medical Center, Amsterdam, The Netherlands; 2 Expertise Center for Palliative Care Amsterdam UMC, Amsterdam, The Netherlands; 3 Viaa University of Applied Sciences, Zwolle, The Netherlands; 4 Department of Medical Oncology, Amsterdam UMC, location VU Medical Center, Cancer Center Amsterdam, Amsterdam, The Netherlands; 5 Department of Anaesthesiology, Amsterdam UMC, location VU Medical Center, Amsterdam, The Netherlands; 6 Department of Intensive Care Medicine, Amsterdam UMC, location VU Medical Center, Amsterdam, The Netherlands; 7 Department of Intensive Care Medicine Adults, Erasmus MC, University Medical Center, Rotterdam, The Netherlands; 8 Foundation Family and patient Centered Intensive Care (FCIC), Alkmaar, The Netherlands; 9 Department of Intensive Care Medicine, Noordwest Ziekenhuisgroep, Alkmaar, The Netherlands

**Keywords:** ICU, Aftercare, Bereavement, COVID-19, Critical care

## Abstract

**Background:**

Bereaved relatives of intensive care unit (ICU) patients are at increased risk of psychological complaints. Aftercare might help them cope with processing the ICU admission and their loved one’s death. There is little (qualitative) evidence on how bereaved relatives experience aftercare. Also, the COVID-19 pandemic likely impacted aftercare provision. We aim to examine how many relatives in Dutch ICUs received aftercare before and during the pandemic and to qualitatively describe their experiences and needs regarding aftercare.

**Methods:**

A mixed-methods study among relatives of patients who died in an ICU before or during the COVID-19 pandemic. Bereaved relatives in six ICUs completed a questionnaire (*n* = 90), including two items on aftercare. These were analyzed using descriptive statistics and Chi-squared tests. Subsequently, both relatives that received and relatives that did not receive aftercare were interviewed about their experiences and needs regarding aftercare. The interviews were thematically analyzed.

**Results:**

After the passing of a loved one, 44% of the relatives were asked by a healthcare professional from the hospital how they were doing, and 26% had had a follow-up conversation. Both happened more often during the first wave of the pandemic than during the second wave or before the pandemic. The most common reason for not having had a follow-up conversation was not knowing about this option (44%), followed by not feeling a need (26%). Regarding the latter, interviewed relatives explained that this would not revive their loved one or that they had already discussed everything they wanted. Relatives who wanted a follow-up conversation, wanted this because this would help them realize the severity of their loved one’s illness, to exchange personal experiences, and/or to thank the ICU team. Those with a follow-up conversation said that they had reviewed the medical course of the admission and/or discussed their (mental) well-being.

**Conclusions:**

ICU healthcare professionals may play a vital role in addressing aftercare needs by asking relatives how they are doing in the weeks following the death of their loved one and offering them a follow-up conversation with an ICU physician. We recommend to include aftercare for bereaved relatives in ICU guidelines.

**Supplementary Information:**

The online version contains supplementary material available at 10.1186/s12904-024-01396-5.

## Background

Approximately one in six patients in the intensive care unit (ICU) dies during their admission [1]. Bereaved relatives of ICU patients may be at high risk for developing psychological complaints, including complicated grief [2, 3]. Aftercare might help bereaved relatives cope with the ICU admission and the death of their loved one, thereby potentially preventing and/or alleviating psychological distress [4].

Multiple studies have investigated what kind of aftercare is provided in ICUs for bereaved relatives by questioning healthcare professionals or ICU managers [4–8]. From these studies we know that aftercare takes different forms and varies, both within ICUs and between ICUs. Among the identified aftercare elements are: immediate condolence meetings following the death [4], opportunities to view the deceased [5], a letter of condolence [5–8], a planned return visit to the ICU [7] and a follow-up meeting with ICU staff in the weeks or months after the death [6–8]. This also shows that the timing of aftercare can vary from within hours to several months after the death. It has been recommended that support for bereaved relatives of ICU patients should extend beyond the immediate period after death [9].

To the best of our knowledge, few studies on aftercare for bereaved relatives of ICU patients have focused on the perspectives and experiences of the relatives themselves, despite the fact that they are the recipients of such care. However, some relatively older studies have quantitatively examined the needs and satisfaction of relatives regarding aftercare showing mixed results. For example, van der Klink et al. (2010) found that 35% of relatives of deceased ICU patients reported a need for a follow-up bereavement service, whereas Downar et al. (2014) reported that 68% of the bereaved relatives wanted to receive support [10, 11]. In addition, Kock et al. (2014) showed that nearly 80% of those who attended a follow-up meeting 4–6 weeks after the death were satisfied with this service [12].

Hence we know that a substantial proportion of bereaved relatives express a desire for aftercare. Furthermore, if provided, such support is generally well received. However, the reasons why relatives of ICU patients want aftercare and the nature of the desired aftercare remain relatively unexplored. Research in other healthcare settings, such as palliative care units, has shown that relatives who receive aftercare generally feel recognized and find the support helpful [13, 14]. Given the risk of psychological distress among bereaved relatives of ICU patients [2, 3], these relatives may be particularly in need of aftercare. In addition, the COVID-19 pandemic might have affected the need for, and provision of aftercare. As mortality rates were significantly higher than usual during the COVID-19 pandemic more relatives were left behind bereaved, especially in the ICU during the first months of the pandemic [15, 16]. Also, the sudden and unpredictable nature of many ICU admissions and deaths during the pandemic [17], may have increased the need and demand for aftercare. However, the high workload and the strict visiting restrictions in the ICU [18] likely complicated the provision of aftercare. Although there were recommendations on how to provide aftercare during the COVID-19 pandemic [19], little is known about how this was done.

Given the paucity of research with first-hand in-depth experiences of relatives regarding aftercare, we aimed to examine how many relatives in Dutch ICUs report having received some form of aftercare, and to further qualitatively describe their experiences and needs regarding aftercare. Additionally, we aimed to investigate whether aftercare during the COVID-19 pandemic differed from the period before the pandemic (pre-COVID-19). This information may help healthcare professionals to tailor aftercare to the needs of bereaved relatives of ICU patients in both pandemic and non-pandemic times.

## Materials and methods

This mixed-methods study had a sequential explanatory design. It combined a retrospective questionnaire study with a subsequent qualitative in-depth semi-structured interview study. This study is part of a larger study among relatives of ICU patients, both discharged and deceased, which also addressed other topics such as important elements of support [20] and treatment decision-making [21]. As the current study is about aftercare for bereaved relatives, only data from relatives of ICU patients who died in the ICU were included. For the questionnaire study, data were collected from first contact persons of ICU patients who died in the ICU both before the COVID-19 pandemic (December 1, 2019 – February 1, 2020), during the first COVID-19 wave (March 15 – May 15, 2020), and during the second wave (October 1, 2020 – January 1, 2021). Next, interviews were conducted with relatives from before COVID-19 and the first COVID-19 wave. Both relatives who had received aftercare and those who had not were included. The results of the questionnaire study were used to develop the topic guide for the interview study. Additionally, the questionnaire data were used to purposively sample the interview participants and to personalize the interviews to some extent. The findings from the interviews were used to interpret and enrich the findings from the questionnaire.

Data were collected in six Dutch ICUs in the northwestern part of the Netherlands. Two ICUs were located in academic hospitals and four in general hospitals. All ICUs were medical ICUs, and four ICUs (two academic and two general) also treated trauma patients. During the first COVID-19 wave, three of the six ICUs used newly developed family support teams, which consisted of non-ICU healthcare professionals, who supported relatives via telecommunication. In the other three ICUs, healthcare professionals from the ICU continued to provide the support themselves, but also via telecommunication [20].

### Aftercare guidelines in the Netherlands and standard practice

The national guideline *Aftercare and rehabilitation of intensive care unit patients* of the Dutch Association for Intensive Care (NVIC) mentions that care for relatives whose loved one died in the ICU is outside the scope of the guideline [22]. Regarding relatives in general, the guideline states that ICU healthcare professionals have a supportive and signaling role, mainly concerning the period of the ICU admission. It is noted that it is desirable to prolong that care task during the aftercare trajectory. According to the guideline, general practitioners (GPs) and primary care in general play a key role in monitoring and treating psychological problems of relatives. In the last sentence on support and aftercare for relatives, it is stated that these professionals also play a crucial role in the care for relatives of deceased ICU patients [22].

The six participating ICUs have aftercare programs for discharged patients that include follow-up conversations about recovery and mental health, return visits to the ICU and meetings with other discharged ICU patients. The organization of aftercare for bereaved relatives is not as extensive and structurally embedded in the ICUs as that for discharged patients. All six ICUs offer bereaved relatives the opportunity to have a follow-up conversation with the attending physician. There is no selection of which relatives qualify for this, but relatives need to make this appointment themselves. The moment when the bereaved relatives are informed about this option varies per ICU: some ICUs mention it during a conversation immediately after the death, while others call the relatives about 6 weeks after the death to inform them about this. Some ICUs also offer the possibility for relatives to visit the ICU if they wish. During the pandemic, most of the follow-up conversations took place by telephone rather than physically in the ICU.

### Questionnaire study population and data collection

Relatives of ICU patients were eligible if they were the first contact person of an adult patient (≥ 18 years) who was admitted to the ICU for three days or more during one of the three designated study periods. Additionally, the patient had to have been supported with invasive mechanical ventilation (pre-COVID-19) or have had a confirmed COVID-19 infection (first and second COVID-19 wave). Insufficient Dutch language proficiency was an exclusion criterion. One relative per patient was allowed to participate (the first contact person).

**Fig. 1:**
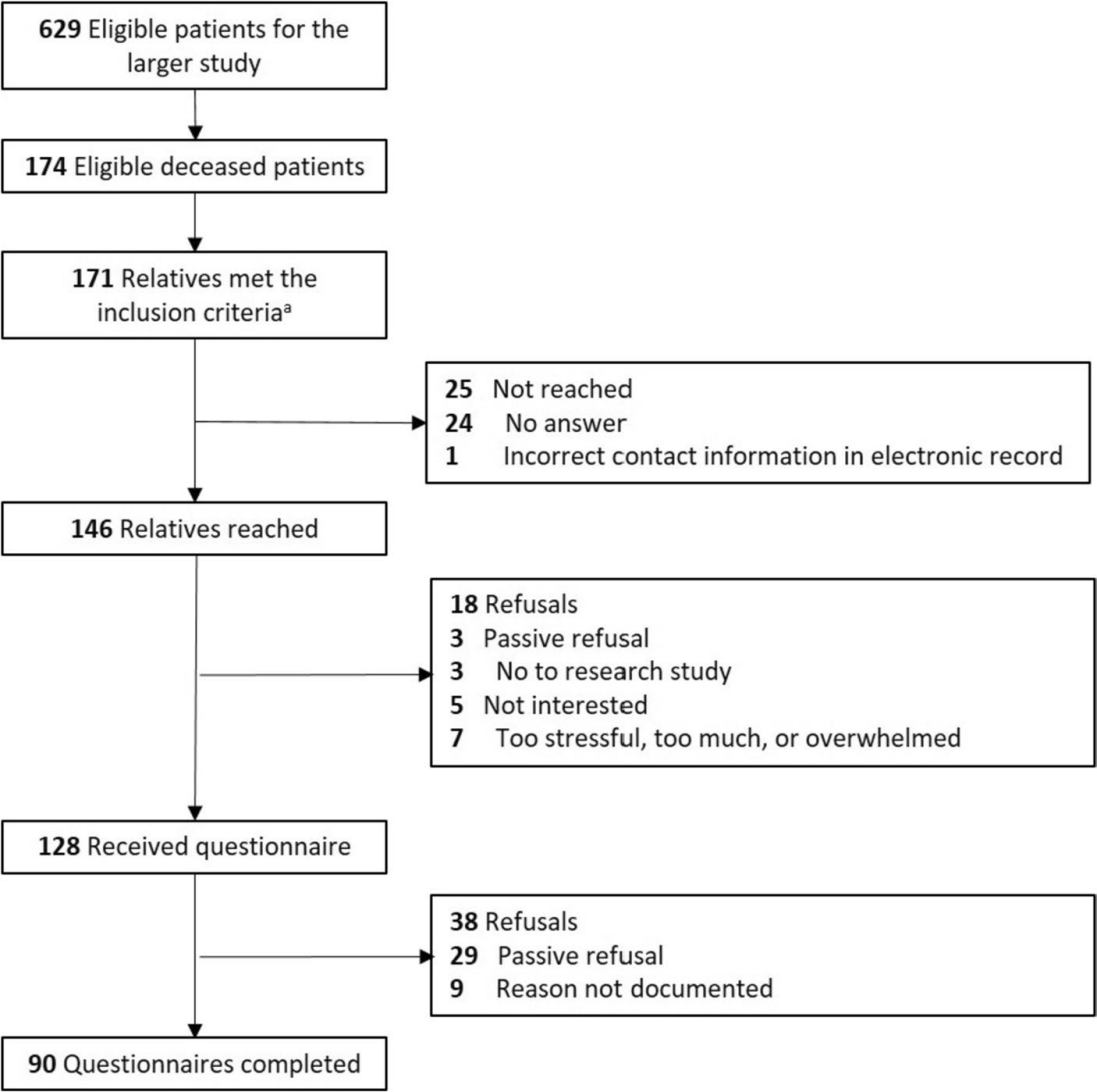
Eligibility and enrolment flowchart. ^a^Four relatives were the first contact persons for two patients in the intensive care unit

The medical records in all six ICUs were searched for patients with the abovementioned criteria using a standardized query developed by one of the researchers (CdP). The resulting records were then manually screened for eligibility by two researchers (CdP and SCR). If the relative was eligible to participate, the contact information was abstracted from the medical record. Relatives from all three study periods were called by one of two researchers (CdP and SCR) about participation in the questionnaire study. The median time between the patient’s ICU admission date and the telephone call was 9.2 months (range 4–18 months). Researchers called relatives a maximum of three times. The call included a short eligibility check, followed by providing verbal study information. If relatives consented to receive the written study information and questionnaire by mail, it was mailed to them within one week. At the beginning of the written questionnaire, relatives were asked to consent to participate in the study. Reminder letters were sent to relatives after three and six weeks if no response was received. In our sample, 171 relatives met the inclusion criteria (Fig. 1). Of these, 146 relatives were reached by telephone. Finally, 90 relatives returned a completed questionnaire (62%).

### Interview study population and data collection

At the end of the questionnaire, relatives were asked if they provided consent to be contacted about participation in an interview. A total of 45 relatives of whom a loved one died in the ICU before COVID-19 or during the first wave indicated that they were willing to be contacted for an interview. Relatives were purposively sampled for the interviews, soughing variation regarding kinship to the patient; gender of the relative; ICU location and whether or not they were supported by a family support team. Of the 15 relatives who were contacted to ask if they would be willing to participate in an interview, 14 relatives participated. The other relative was not available by phone. Among the interviewed relatives there were both relatives who did receive aftercare and relatives who did not receive aftercare. The interviews were conducted by four researchers (SCR, ZJ, CdP and HTK) between May and September 2021, which was on average 15 months (12–20 months) after the death of their loved one. All researchers were trained in qualitative interviewing and had no prior relationship with the relatives. Due to the COVID-19 restrictions, interviews were conducted by telephone (*n* = 11) or video call (*n* = 3), depending on the relative’s preference. The interviews lasted on average 45 min (15–65 min) and were audio-recorded.

### Questionnaire measurements

The questionnaire included questions on the characteristics of relatives and patients and on (experiences with) support that relatives received during the ICU admission (Additional file 1). The variables of interest for the present study are the demographic characteristics of relatives and patients and two items on aftercare in the ICU (Additional file 1, questions 50 & 51). Demographic characteristics included kinship, age, gender, level of education and cultural background of the relative and gender and age of the patient. The two items on aftercare were: (1) did a healthcare professional from the hospital ask you in the weeks/months after the death of your loved one how you were doing? (yes/no); (2) did you have an appointment with an ICU physician after the death of your loved one (e.g. to discuss the ICU admission)? (yes/no). For the latter item, the “no” category consisted of several options according to the reason for not having had an appointment.

### Interview topic list

The interviews were guided by a semi-structured topic list related to experiences with support in the ICU (Additional file 2). The interview topics that are of interest for the current study are relatives’ experiences with aftercare and their needs regarding aftercare. Questions were tailored, if possible, to specific responses in the questionnaire (e.g. “In the questionnaire you mentioned that you did not know about the possibility of aftercare. To what extent would you have liked to have received some form of aftercare?).

### Questionnaire analysis

IBM SPSS Statistics 26 was used for statistical analyses. Descriptive statistics were used to describe the sample characteristics and relatives’ experiences with aftercare, both for the total population and for relatives from the three study periods separately. Differences between relatives from the three study periods were tested using chi-squared tests for categorical data. Chi-squared tests were replaced by Fisher’s exact tests when > 20% of the cells in a contingency table had an expected count of less than five.

### Interview analysis

All interviews were transcribed verbatim and analyzed in MAXQDA following the principles of thematic analysis through the “six-phase” approach developed by ﻿Braun and Clarke [23, 24]. First, the transcripts were read multiple times before coding. The transcripts of the first three interviews were coded inductively by two researchers (SCR and ZJ) and extensively discussed by four researchers (SCR, ZJ, HRP and BOP). After discussion, some codes were refined. Subsequently, all remaining transcripts were coded by SCR using the refined codebook. The codes were then grouped into themes by three researchers (SCR, HRP and BOP). After coding 14 interviews, it seemed that inductive thematic saturation had been reached, as no new codes and themes emerged from the data [25, 26]. Therefore we concluded that no additional interviews were needed. The themes were discussed and grouped within the research team. The research group consisted of researchers with different backgrounds (health sciences, medical anthropology, sociology, social psychology), as well as physicians and nurses. Finally, the quotations were translated by a professional translator and checked by a second professional translator.

### Ethics

Ethical approval was waived for this study by the Medical Ethics Review Committee of VU University Medical Center because the study is not subject to the Dutch Medical Research Involving Human Subjects Act (registration number 2020.0618). Additionally, institutional review boards at each site approved all procedures (Dijklander Science Centre and Board of Directors Dijklander Ziekenhuis (DOC 020), Board of Directors Ziekenhuis Amstelland (n.s.), Board of Directors Zaans Medisch Centrum (HF21038), Science Office Noordwest Ziekenhuisgroep (L021-037)). The relatives were informed orally and in writing about the questionnaire and interview. Before filling in the questionnaire, relatives provided written informed consent. Data were pseudonymized. If relatives wished to participate in an interview they were asked to provide their e-mail address and/or telephone number at the end of the questionnaire. Before the interview, relatives gave oral informed consent. After transcription, the audio recordings were deleted and the transcripts were anonymized to protect the privacy of the participants. All methods were performed in accordance with the relevant guidelines and regulations of the Declaration of Helsinki and with the Netherlands Code of Conduct for Scientific Practice of the Association of Universities in the Netherlands (VSNU).

## Results

The 90 relatives who filled in the questionnaire were mostly the patient’s partner (50.0%) or the son/ daughter (37%), female (70.0%), 51 years or older (70.0%), medium or highly educated (82.0%), and had a Dutch cultural background (92.2%) (Table 1). The admitted patients were mostly men (68.9%), 66 years or older (71.1%) and stayed in the ICU for 11 days or longer (57.8%). Relatives from the second wave had significantly less often a Dutch cultural background, and more often another cultural background (e.g. Surinamese or Moroccan).

**Table 1:** Relative and patient demographic characteristics from the questionnaire (absolute numbers and rounded percentages)

			Pre-COVID-19 (*n* = 25)	First COVID-19 wave (*n* = 39)	Second COVID-19 wave (*n* = 26)	Total (*n* = 90)	*p*-value
*Relative characteristics*							
Kinship to patient							0.755
Partner			14 (56.0%)	20 (51.3%)	11 (42.3%)	45 (50.0%)	
Son/daughter			7 (28.0%)	15 (38.5%)	11 (42.3%)	33 (36.7%)	
Other			4 (16.0%)	4 (10.3%)	4 (15.4%)	12 (13.3%)	
Gender							0.202
Male			11 (44.0%)	9 (23.1%)	7 (26.9%)	27 (30.0%)	
Female			14 (56.0%)	30 (76.9%)	19 (73.1%)	63 (70.0%)	
Age							0.317
< 30 years			0	1 (2.6%)	0	1 (1.1%)	
30–50 years			4 (16.0%)	14 (35.9%)	8 (30.8%)	26 (28.9%)	
51–65 years			15 (60.0%)	13 (33.3%)	13 (50.0%)	41 (45.6%)	
66 years or older			6 (24.0%)	11 (28.2%)	5 (19.2%)	22 (24.4%)	
Level of education							0.709
Low			6 (25.0%)	6 (15.4%)	4 (15.4%)	16 (18.0%)	
Medium			11 (45.8%)	15 (38.5%)	11 (42.3%)	37 (41.6%)	
High			7 (29.2%)	18 (46.2%)	11 (42.3%)	36 (40.4%)	
Cultural background^b^							
Dutch			24 (96.0%)	38 (97.4%)	21 (80.8%)	83 (92.2%)	**0.047**
Other^a^			1 (4.0%)	2 (5.1%)	6 (23.1%)	9 (10.0%)	**0.035**
*Patient characteristics*							
Gender							0.074
Male			14 (56.0%)	26 (66.7%)	22 (84.6%)	62 (68.9%)	
Female			11 (44.0%)	13 (33.3%)	4 (15.4%)	28 (31.1%)	
Age							0.135
< 30 years			0	1 (2.6%)	0	1 (1.1%)	
30–50 years			1 (4.0%)	2 (5.1%)	0	3 (3.3%)	
51–65 years			8 (32.0%)	7 (17.9%)	7 (26.9%)	22 (24.4%)	
66 years or older			16 (64.0%)	29 (74.4%)	19 (73.1%)	64 (71.1%)	
ICU length of stay							0.071
3–5 days			2 (8.0%)	1 (2.6%)	0	3 (3.3%)	
5–10 days			13 (52.0%)	16 (41.0%)	6 (23.1%)	35 (38.9%)	
11–20 days			6 (24.0%)	11 (28.2%)	15 (57.7%)	32 (35.6%)	
> 20 days			4 (16.0%)	11 (28.2%)	5 (19.2%)	20 (22.2%)	

Missing values: education 1

^a^ e.g. Surinamese and Moroccan

^b^: multiple answers possible

The 14 relatives who were interviewed were mainly female (*n* = 9), the patient‘s partner (*n* = 9) or their son/ daughter (*n* = 5), and the ICU admission occurred in most cases during the first COVID-19 wave (*n* = 9) (Table 2).

**Table 2:** Characteristics of relatives participating in interviews

	COVID-19 period	Gender	Kinship	Gender patient	Age patient	Family support team
1	First wave	Female	Partner	Male	66–80 years	No
2	First wave	Male	Partner	Female	66–80 years	Yes
3	First wave	Female	Daughter^a^	Male	51–65 years	Yes
4	Pre-COVID-19	Female	Daughter^a^	Male	80 + years	N/A
5	First wave	Male	Son^a^	Female	66–80 years	Yes
6	Pre-COVID-19	Male	Partner	Female	66–80 years	N/A
7	First wave	Female	Partner	Male	51–65 years	Yes
8	First wave	Female	Partner	Male	66–80 years	Yes
9	First wave	Female	Daughter^a^	Female	66–80 years	No
10	Pre-COVID-19	Female	Partner & daughter^a,b^	Male	51–65 years	N/A
11	Pre-COVID-19	Male	Partner	Female	51–65 years	N/A
12	First wave	Female	Partner	Male	66–80 years	No
13	Pre-COVID-19	Male	Partner	Female	80 + years	N/A
14	First wave	Female	Daughter	Male	66–80 years	No

^a^Children in law are also included in this category

^b^Interview with both the partner and the daughter of a patient. The partner was the registered first contact person of the patient

Overall 43.9% of the relatives reported that in the weeks or months following the death of their loved one, a healthcare professional from the hospital had asked how the relative was doing (Table 3). Significantly more relatives from the first COVID-19 wave reported having been asked how they were doing (60.5%), compared with relatives from pre-COVID-19 (35.0%) and the second wave (25.0%) (*p* = 0.014). In addition, 26.2% of the relatives reported that they had had an appointment with an ICU physician after the death of their loved one, for example to review the ICU admission. The 73.8% of relatives who reported they did not have an appointment with an ICU physician gave several reasons for this. The most common reason was that they had not seen, read or heard about the possibility of making an appointment (43.5%). Other reasons were that relatives did not feel the need for aftercare (25.8%), that it was not allowed due to COVID-19 restrictions (10.9%), and that they feared that going back to the hospital would relive negative experiences (6.5%). A few relatives also mentioned that the ICU was too far away, that there was no point in making an appointment because it would not revive their loved one, that they already had had an appointment with another non-ICU physician in the hospital, and that the relative did not want to bother the ICU physician with such an appointment (during COVID-19).

**Table 3:** Aspects of aftercare for relatives of ICU patients

	Pre-COVID-19 (*n* = 25)	First wave (*n* = 39)	Second wave (*n* = 26)	Total (*n* = 90)	*p*-value
Did a healthcare professional from the hospital ask you in the weeks/months after your loved one died how you were doing? Yes	7 (35.0%)	23 (60.5%)	6 (25.0%)	36 (43.9%)	0.014
Did you have an appointment with an ICU physician after the death of your loved one?^e^					
Yes	5 (23.8%)	14 (35.9%)	3 (12.5%)	22 (26.2%)	0.128
No, namely.^a^	16 (76.2%)	25 (64.1%)	21 (87.5%)	62 (73.8%)	
	***n = 16***	***n = 25***	***n = 21***	***n = 62***	
… due to COVID-19 restrictions^b^	NA	5 (20.0%)	0	5 (10.9%)	0.054^c^
… no need	5 (31.3%)	6 (24.0%)	5 (23.8%)	16 (25.8%)	0.871
… did not know about option	5 (31.3%)	12 (48.0%)	10 (47.6%)	27 (43.5%)	0.555
… possible relive of negative experiences	0	2 (8.0%)	2 (9.5%)	4 (6.5%)	0.669^c^
… other reason^d^	6 (37.5%)	5 (20.0%)	7 (33.3%)	18 (29.0%)	0.406

^a^The percentages for the different reasons why relatives had not had an appointment with an ICU physician is calculated as a proportion of the people who did not have an appointment (resp. 16, 25, 21 and 62 relatives)

^b^Only asked to relatives from the first and second COVID-19 wave

^c^Fisher’s exact test instead of a chi-squared test, because > 20% of the cells had an expected count of less than 5

^d^Examples of other reasons mentioned: relative felt that the ICU was too far away; according to the relative it makes no sense because you do not get your loved one back through the appointment; relative does not want to bother the ICU physician

^e^Multiple answers possible

More relatives from the first wave had had an appointment with an ICU physician (35.9%) than relatives from pre-COVID-19 (23.8%) and the second wave (12.5%), but this was not statistically signficant different. The reasons for not having had an appointment also did not differ significantly between the three study periods.

### Is there a need for ICU aftercare?

In the interviews both relatives who had and relatives who had not received aftercare explained why they did or did not feel a need for aftercare. One of these reasons was that reviewing the ICU admission with the healthcare professionals involved and being able to ask questions increased awareness of the severity of the loved one’s illness:

“*And that [follow-up conversation about the autopsy results] did give me more peace of mind like okay, he’s passed away and he was really sick. He just made himself out to be stronger than he really was.”* (4: daughter, pre-COVID-19).

*“I would like to hear an explanation based on the scan: how severe was it exactly, what did it look like? And to have an image to go with the story in my mind, and that I can put it into realistic perspective in terms of the severity, yes. To really be able to process how intense it all was.”* (10: daughter, pre-COVID- 19).

Other relatives also indicated that an appointment with the ICU healthcare professionals provides an opportunity to ask questions about developments in the patient’s medical situation during the admission. In addition, relatives mentioned that a follow-up conversation would allow them to share their own experiences of the admission, but also for the healthcare professionals to share their experiences and feelings about the patient’s ICU admission:

*“Discuss things, maybe ask things like how could it all happen so fast. And is there a cause… well, there was a cause, but what does that mean, were there no other options? Things like that. Okay, it was deteriorating but was it really that bad? Yes, it really was that bad. Maybe you’d try to tell your story, and he [the physician] could also get it off his chest. Because I assume they are human too, so it’s not always going to be easy for them either.”* (11: partner, male, pre-COVID-19).

Some relatives mentioned that they would have liked to express their gratitude to the ICU healthcare professionals for the care and support they received, for example in a follow-up conversation and/or on a return visit to the ICU.

Relatives who had had a follow-up conversation with ICU healthcare professionals were happy with this conversation and found it valuable:

*“So at some point I got a letter I think, saying I was invited for one of these conversations. And it was just in person again with the doctor. And it was that one doctor who saw him a lot in the beginning. So he’d seen him in the ward, and the ICU doctor. So he had really experienced all of it. And the nurse was there too. And that was really good. They ran through everything again and at the end they asked, ‘Well, do you have anything we could learn from? What could we have done better?’ So that was a really good conversation.”* (8: partner, female, first wave).

One relative who had not been contacted by a healthcare professional from the hospital to ask how he was doing after his partner’s death, was very upset about this. He felt it was inhumane:

I: “*How did you feel, that you weren’t asked about that [how are you doing]?*”

R: “*It felt to me like it’s some kind of factory. You know, out of sight is out of mind. That one’s gone and we’re moving on. And I do understand that in a way, I can empathize with that to a certain extent, of course I can. But after she passed away… the only one who called was the man who wanted money for opening up [access to the mortuary in the weekend] and I didn’t hear from anyone else. […] There is just a complete lack of any personal touch.*” (2: male, partner, first wave).

Among the relatives who had had a follow-up conversation, some emphasized that they highly valued having this conversation with the ICU physician with whom they had had the most contact during the ICU admission. According to these relatives, they can then speak first-hand during the conversation, and there is already some kind of connection:

R: “*So when my father passed away we had a conversation with the doctor and a nurse. And half a year later we had that same conversation with a doctor and a nurse. […] And I thought that was really nice.”*

I: “*Is that what made it so nice, that it was the same as during that last conversation?*”

R: “*Yes… yes, maybe that too… perhaps it sounds a bit strange, but to really get closure together. Because this doctor was there right up to the end, when my father passed away, he was also the doctor who did the autopsy, and so he was there to discuss the autopsy results as well. And well, I … well because then you already … you have a bit of a bond with someone then. This doctor was also involved in the conversation the first night, when my father had surgery, he was there too. So yes… and maybe it’s a coincidence, but I thought it was really nice. […] What I especially liked is that he did the autopsy himself, so he was really talking based on his own findings.*” (4: daughter, pre-COVID-19).

Some relatives who had not yet had a conversation with an ICU physician, said at the time of the interview that they would still appreciate this, while others would have liked it shortly after the admission but would not like it anymore.

*“So […] I think, well I wouldn’t mind still having that conversation.”* (10: daughter, pre-COVID-19).

*“Maybe I look at it a bit differently now. Maybe at the time, if that had been the case. […] Not anymore now, no.”* (11: partner, male, pre-COVID-19).

On the other hand, several relatives did not feel the need for aftercare. None of them received it. They gave two main reasons for not wanting aftercare. First, some relatives explained that aftercare would not change the outcome of the admission, namely the death of their loved one. Therefore, they saw no point in reviewing the ICU admission with healthcare professionals:

*“Well, more when there was still some point to it, when it could still have meant that he might get better. I had more conversations with the doctor then. But after his death, I didn’t have that need to talk about it again and how it really came about and what he actually died from — right, the coronavirus.”* (7: partner, female, first wave).

The second reason given by some of the relatives who did not feel the need for aftercare was that they had already discussed everything they wanted to discuss during the ICU admission and immediately after the death of their loved one. These relatives did not know what else they could discuss if they had a conversation with healthcare professionals in the weeks or months following the ICU admission:

*“Then she [ICU doctor] said, ‘do you want to talk to us again?’ […] Well, I thought we had just discussed… I don’t know what else I should be discussing with her other than that. So I said, ‘well no, no need’. […] I thought: what else am I supposed to say about it to this woman? I mean… I don’t know, a brain haemorrhage and now he’s gone. As for the rest, I don’t know either. So we didn’t do that.”* (1: partner, female, first wave).

Finally, one relative indicated that she was unsure if she would have wanted aftercare because she did not get to experience it.

### What is discussed with healthcare professionals in follow-up conversations?

In the interviews, relatives mentioned two main topics that were discussed in follow-up conversations with healthcare professionals. First, relatives indicated that healthcare professionals explained and clarified medical events that had occurred during the ICU admission and reviewed this period together. In several cases, autopsy was performed and the results were discussed with the relatives by the ICU physician:

*“An autopsy was performed on my mother-in-law’s body; they asked permission for that and got it. So we talked about what came out of that. And at that time a bit more was becoming known about the treatment of COVID, so they also referred to that and what exactly happened and yes, what the cause could have been.”* (5: son-in-law, male, first wave).

As mentioned earlier, relatives indicated that discussing the medical course can help them realize the seriousness of the situation and the inevitability of their loved one’s death.

The second topic that was discussed in the aftercare conversations according to several relatives was their well-being, especially their mental well-being. Most of them greatly appreciated the attention paid to this by the ICU healthcare professionals:

*“I also thought that they were very good about taking plenty of time for that [topics other than medical matters]. Because we talked for at least one hour, with my brother and my husband there again, with the doctor and the nurses. Well, I’m pretty comfortable talking about my feelings, but my brother isn’t. At some point the doctor noticed that too. So he specifically asked my brother, ‘How are you doing’? Because I’d started off talking about how I’m doing, really the personal stuff… There was plenty of room for that. And well, like I said, my brother isn’t very good at talking about his feelings, so the doctor asked him, ‘Hey, how are you doing’?”* (4: daughter, pre-COVID-19).

One relative also mentioned that the attending ICU physician and an ICU nurse informed her about the post-intensive care syndrome-family in their conversation:

*“They asked about that [how are you]. […] They explained that relatives of ICU patients can get post-traumatic stress disorder. And their family members too. So they asked how I was doing, if I thought it was affecting me and how [son’s name] was doing.”* (8: partner, female, first wave).

Another relative said that she and her family were also asked how they were doing, but that she felt it was more of a formality than genuine interest:

R: *“So other than the information about the examination of my father after his death, she didn’t really tell us much.”*

I: *“And during that conversation there was no room for you and how you were doing, if I understand you correctly?”*

R: *“Well, if I have to be honest, of course she did briefly ask how we were doing, but more as a sign of respect.”* (3: daughter, first wave).

### Role of general practitioners in aftercare

Several relatives reported that they did not receive aftercare from the ICU, but instead from their GP. In some cases, this was due to the unavailability of ICU healthcare professionals during the first months of the COVID-19 pandemic. One relative explained that physicians from the hospital provided the opportunity for a conversation to get more information about the cause of death, but that they could also get this information through the GP:

*“The internal medicine specialists had also said, ‘If you would like to talk afterwards so you know what she died of — she died of the coronavirus, but what exactly was behind it all’? And so on. ‘We can explain it in layman’s terms afterwards, but you can also do that with your GP’. So the three of us went to the GP.”* (9: daughter, first wave).

This was the only relative who mentioned discussing medical details of the ICU admission with the GP. Other relatives said that the aftercare they received from their GP concerned emotional support and regular checks on how they were doing:

*“The GP did tell us multiple times that if we needed psychological help later, she would absolutely support us in that. So there was that. She also calls… mainly my mother, every now and then, once every three months or so, to ask how things are going. So there’s all that, and we feel very supported by the GP.”* (3: daughter, first wave).

*“The GP did tell us multiple times that if we needed psychological help later, she would absolutely support us in that. So there was that. She also calls… mainly my mother, every now and then, once every three months or so, to ask how things are going. So there’s all that, and we feel very supported by the GP.”* (2: male, partner, first wave).

### Provision of aftercare impacted by COVID-19 restrictions

Findings from the interviews with relatives from pre-COVID-19 and the first wave confirmed that aftercare was hindered by the COVID-19 restrictions. As aftercare usually takes place in the weeks or months following a patient’s death in the ICU, relatives of patients who died before COVID-19 (between December 2019 and February 2020) also mentioned that they were faced with the COVID-19 restrictions. Some relatives indicated that because of these restrictions, the aftercare took place later than they had hoped and/or planned for:

“*And well, then we had the coronavirus, so it took a little bit longer than planned. But I think that it was half a year later maybe, when we had another talk at the hospital.*” (4: daughter, pre-COVID-19).

Relatives understood that aftercare was delayed due to the pandemic. A few relatives mentioned that they did not want to bother the healthcare professionals during these busy times in the ICU. As two relatives of a patient who died two months before the COVID-19 outbreak explained:

R: *“In the end we did want to have that conversation… but well then the coronavirus pandemic suddenly started…”*.

R2: *“Yes, it was offered to us, as in if in a few months you’d like to know what exactly was wrong and if you have any questions, you’re always welcome to have a chat with one of the doctors. But then the coronavirus broke out and of course the whole of the Netherlands was in chaos. […] Well, we kind of laid it to rest and thought well, they have other things on their mind right now rather than talking to us.*” (10: partner (R) & daughter (R2), female, pre-COVID-19).

## Discussion

This mixed-methods study provides valuable insights into the experiences and needs of bereaved relatives of ICU patients for aftercare. Less than half of these relatives were asked by healthcare professionals about their well-being shortly after their loved one’s death and approximately a quarter had a follow-up conversation with an ICU physician. Notably, during the first COVID-19 wave, a higher percentage of relatives received aftercare compared with both the pre-COVID-19 period and the second wave. The interviews revealed varying needs for aftercare among relatives. Some did not feel the need for it, because aftercare would not bring their loved one back to life and/or everything had already been discussed. Others wanted aftercare to help them understand the severity of their loved one’s illness, exchange personal experiences, and/or to thank the ICU team. Topics discussed in follow-up conversations included the medical course of the ICU admission and the relatives’ mental well-being. Relatives particularly appreciated follow-up conversations with the ICU physician who had been most involved during the admission. Finally, in some cases, GPs also played a role in providing aftercare.

### A limited number of relatives receive aftercare

Across all study periods, only a limited number of bereaved relatives received aftercare from the ICU. This resonates with a Swedish study conducted before the pandemic, focusing on hospital deaths in general, which found that a quarter of the relatives had had a follow-up conversation [14]. In our study, nearly 26% of the relatives who had not had a follow-up conversation said that they felt no need to have one. This may give the impression that the limited use of aftercare services is due to a perceived low need among the bereaved. However, the largest proportion of relatives who had not been offered a follow-up conversation reported that the main reason for not seeking aftercare was that they were not aware of the possibility of such an appointment. These findings suggest a lack of communication between ICU healthcare professionals and relatives about the availability and potential benefits of aftercare. ICU healthcare professionals may need to be made more aware of the importance of informing relatives about aftercare options. Also we recommend ICU healthcare professionals to offer aftercare to all relatives, instead of assessing themselves who will or will not need aftercare. Relatives can then decide for themselves whether they would like to make use of it. Aftercare can include elements that we have studied, such as a short conversation about how a relative is doing shortly after the death as well as a longer follow-up conversation with an ICU physician after several weeks or months. Other options such as sending a letter of condolence and an ICU return visit may also be considered [5–8].

### Need for reviewing the medical course and for attention for own well-being

Many relatives express a desire for aftercare, particularly a follow-up conversation. The reasons for this are similar to those found by van der Klink et al. (2010), such as asking questions that were not previously asked, asking new questions that have arisen during the bereavement period, receiving support in coping with the loss of a loved one, and wanting to thank the ICU staff [10]. Similar to what we found in the ICU, research by Milberg et al. (2008) in palliative care units showed that bereaved relatives wanted to review what had happened during the palliative phase [13].

Some relatives specifically indicated that they highly valued reviewing the ICU admission with the healthcare professional who was most involved in the treatment and decision-making, because they felt a sense of connection with this person. Likewise, Milberg et al. (2008) found that having a follow-up meeting with a healthcare professional with whom the relative has an established relationship instilled feelings of security and trust among bereaved relatives [13]. In particular, these relatives expressed a desire to speak with a healthcare professional who had visited the patient in the final hours before death.

Additionally, previous research has shown that some relatives feel the need to discuss their current well-being [13]. This was not a primary reason for wanting aftercare among our study participants. However, it was highly appreciated by relatives who had discussed their well-being in a follow-up conversation. This highlights the importance of healthcare professionals actively addressing relatives’ well-being, as relatives may not naturally raise the topic themselves. Based on these findings, we recommend that aftercare activities focus on discussing the events that led up to the patient’s death and leave room for questions, and addressing the relatives’ current well-being (beyond a polite “how are you” as a conversation starter). In general, ICU clinicians appear to feel reasonably comfortable discussing the medical details of the patient’s admission, as well as relatives’ well-being [11]. However, some physicians may feel unequipped to address (severe) psychological distress, such as complicated grief. This reluctance was evident in a study in the general hospital setting. Some healthcare professionals did not feel competent enough to provide bereavement care and one in four physicians did not even consider this part of their role [27]. Regarding the latter, the National Dutch ICU guideline states that ICU healthcare professionals have a supportive and signaling role towards relatives [22]. Yet, the guideline does not cover the period following the patient’s death. Lack of perceived competence or responsibility may be a barrier to the necessary and appreciated aftercare. ICU physicians should however be aware that they are not the designated healthcare professionals to treat relatives with psychological complaints. Nonetheless, by making inquiries on relatives’ well-being, ICU physicians can identify those who need additional support and suggest that they contact appropriate resources, such as their GP.

### The extraordinary situation during the first COVID-19 wave enhanced aftercare

Considering the extraordinary situation during the first months of the pandemic, especially with the high workload for healthcare professionals, it seems counterintuitive that we found the highest percentage of relatives being asked about their well-being by healthcare professionals during this period (60.5% in the first wave versus 35.0% pre-COVID-19 and 25.0% in the second wave). This suggests that during this period healthcare professionals may have become increasingly aware of the pandemic’s psychological impact on bereaved relatives, leading them to pay more attention to the relatives’ mental well-being. Furthermore, due to rigorous visiting restrictions, healthcare professionals may have felt a greater sense of urgency to reach out to relatives, as they did not have regular face-to-face interactions. Similarly, more relatives from the first wave had a follow-up conversation with an ICU physician (35.9% versus 23.8% pre-COVID-19 and 12.5% second wave), but this difference was not statistically significant. Interestingly, the reasons for not having a follow-up conversation with an ICU physician did not differ between study periods. For example, the proportion of relatives who did not feel the need for such an appointment, or who were unaware of the possibility of having a follow-up appointment did not differ significantly. This suggests that the barriers to accessing aftercare persist regardless of the contextual factors.

### Practical implications

Although a considerable proportion of relatives would like to receive aftercare after the death of their loved one, only a limited number of relatives receive it. As the topic of aftercare for bereaved relatives is not yet included in the Dutch national guideline on ICU aftercare and is also uncommon internationally [5], we recommend that it is considered for inclusion in ICU guidelines. This should include information such as what aftercare entails, who is responsible for this, who should the aftercare be directed to, and when this aftercare should be provided. Suggestions for core components of this guideline on aftercare for bereaved relatives of ICU patients based on our results can be found in Table 4. Such a guideline has the potential to achieve a more structural embedding of aftercare for bereaved relatives in ICUs. However, this requires that the guideline is known to healthcare providers and that ICU healthcare providers are aware of the importance of this aftercare and their responsibilities in this regard. Also organizations in which the healthcare providers works need to facilitate the implementation of aftercare. Furthermore, there are and will be barriers to the implementation of aftercare. Therefore, it is important to examine what barriers exist to providing aftercare and how these can be addressed. For example, ICU healthcare professionals do not have a legal treatment relationship with relatives [22], which in turn may affect the funding of aftercare and create limitations in referring relatives to appropriate care. Furthermore, the division of roles and responsibilities between ICU healthcare professionals and primary care, including GPs, should be further explored. While ICU healthcare professionals seem to be the appropriate person to review the medical course of the ICU admission, GPs may be able to take the lead in discussing mental well-being with relatives.

**Table 4:** Recommendations for core components of an ICU guideline on aftercare for bereaved relatives

• We suggest that aftercare should minimally include asking how the bereaved relative is doing in the weeks or months after the death, and a more elaborate follow-up conversation;	
• In the follow-up conversation discuss both the medical details of the ICU admission and the mental well-being of the bereaved relatives;	
• ICU healthcare professionals provide the aftercare, and the follow-up conversation is preferably led by the physician who was most involved in the care during the ICU admission;	
• Consider collaboration with GPs and primary care in the provision of aftercare, e.g. a leading role in the emotional and bereavement support;	
• Give all relatives the opportunity to receive aftercare. Make no selection of relatives who are eligible. Relatives themselves can decide whether they want it or not;	
• We suggest that aftercare is offered at multiple times. An ICU healthcare professional explains the aftercare possibilities shortly after the patient’s death. This should be repeated at a later time, e.g. 4-6 weeks later, as relatives may not be able to process this information properly at this stressful time and their needs may change over time.	

### Strengths and limitations

This study has several strengths and limitations that must be considered when interpreting the results. One of the strengths is the sequential explanatory mixed-methods design. This design allows for a comprehensive exploration of bereaved relatives’ experiences with aftercare provision in the ICU. Furthermore, to our knowledge, this study is one of the first to conduct qualitative in-depth interviews with relatives about aftercare. A potential limitation is the risk of recall bias. The median time between the patient’s ICU admission and telephone contact for participation was 9.2 months. This may have affected the accuracy of participants’ recall, e.g. they may have forgotten that a healthcare professional called to ask how they were doing. However, in the interviews relatives described very detailed experiences and feelings, suggesting that they were still well able to recall the ICU admission and the period following the death of their loved one. Additionally, due to the COVID-19 restrictions all interviews were conducted by telephone or video call. This may have made it more difficult to build rapport- with relatives, resulting in potentially less in-depth interviews. However, several relatives expressed appreciation for the fact that the interview was not face-to-face, as they felt more comfortable sharing their experiences over a telephone or video call.

## Conclusion

To conclude, bereaved relatives of ICU patients often go through stressful experiences during the ICU admission and following the death of their loved one, making aftercare important for their support. However, numerous relatives do not receive aftercare, mainly due to a lack of information from healthcare professionals about the options available. Many bereaved relatives wish to have a follow-up conversation to review the medical course of the ICU admission and place a high value on discussing their well-being, preferably with the physician most closely involved. To strengthen clinical practices, we recommend that aftercare for bereaved relatives is included in local or (inter)national ICU guidelines.

### Supplementary Information

**Supplementary Material 1.**

**Supplementary Material 2.**

## Data Availability

The data that support the findings of this study are available from the corresponding author, SCR, upon reasonable request.

## Abbreviations

ICU: Intensive care unit
GP: General practitioner

## References

[1] Vincent J-L, Marshall JC, Ñamendys-Silva SA, François B, Martin-Loeches I, Lipman J (2014). Assessment of the worldwide burden of critical illness: the Intensive Care Over Nations (ICON) audit. Lancet Respiratory Med.

[2] Kentish-Barnes N, Chaize M, Seegers V, Legriel S, Cariou A, Jaber S (2015). Complicated grief after death of a relative in the intensive care unit. Eur Respir J.

[3] Naef R, von Felten S, Ernst J (2021). Factors influencing post-ICU psychological distress in family members of critically ill patients: a linear mixed-effects model. Biopsychosoc Med.

[4] Kentish-Barnes N, Chevret S, Valade S, Jaber S, Kerhuel L, Guisset O (2022). A three-step support strategy for relatives of patients dying in the intensive care unit: a cluster randomised trial. Lancet.

[5] Egerod I, Kaldan G, Albarran J, Coombs M, Mitchell M, Latour JM (2019). Elements of intensive care bereavement follow-up services: a European survey. Nurs Crit Care.

[6] Efstathiou N, Walker W, Metcalfe A, Vanderspank-Wright B (2019). The state of bereavement support in adult intensive care: a systematic review and narrative synthesis. J Crit Care.

[7] Egerod I, Kaldan G, Coombs M, Mitchell M (2018). Family-centered bereavement practices in Danish intensive care units: a cross-sectional national survey. Intensive Crit Care Nurs.

[8] Moss SJ, Wollny K, Poulin TG, Cook DJ, Stelfox HT, Ordons ARd, Fiest KM (2021). Bereavement interventions to support informal caregivers in the intensive care unit: a systematic review. BMC Palliat Care.

[9] Eskell M, Thompson J, Powell O, Torlinski T, Mullhi R (2022). Understanding the Intensive Care Unit experience of patients and relatives at the end-of-life during the Coronavirus Disease 2019 Pandemic. J Patient Experience.

[10] van der Klink MA, Heijboer L, Hofhuis JGM, Hovingh A, Rommes JH, Westerman MJ, Spronk PE (2010). Survey into bereavement of family members of patients who died in the intensive care unit. Intensive Crit Care Nurs.

[11] Downar J, Barua R, Sinuff T (2014). The desirability of an Intensive Care Unit (ICU) clinician-led bereavement screening and support program for family members of ICU decedents (ICU bereave). J Crit Care.

[12] Kock M, Berntsson C, Bengtsson A (2014). A follow-up meeting post death is appreciated by family members of deceased patients. Acta Anaesthesiol Scand.

[13] Milberg A, Olsson E-C, Jakobsson M, Olsson M, Friedrichsen M (2008). Family Members’ Perceived needs for Bereavement Follow-Up. J Pain Symptom Manag.

[14] O’Sullivan A, Alvariza A, Öhlén J, Larsdotter C (2021). Support received by family members before, at and after an ill person’s death. BMC Palliat Care.

[15] Richards-Belle A, Orzechowska I, Gould DW, Thomas K, Doidge JC, Mouncey PR (2020). COVID-19 in critical care: epidemiology of the first epidemic wave across England, Wales and Northern Ireland. Intensive Care Med.

[16] Dongelmans DA, Termorshuizen F, Brinkman S, Bakhshi-Raiez F, Arbous MS, de Lange DW (2022). Characteristics and outcome of COVID-19 patients admitted to the ICU: a nationwide cohort study on the comparison between the first and the consecutive upsurges of the second wave of the COVID-19 pandemic in the Netherlands. Ann Intensive Care.

[17] Filipovic N, Saveljic I, Hamada K, Tsuda A (2020). Abrupt deterioration of COVID-19 patients and spreading of SARS COV-2 virions in the lungs. Ann Biomed Eng.

[18] Tabah A, Elhadi M, Ballard E, Cortegiani A, Cecconi M, Unoki T (2022). Variation in communication and family visiting policies in intensive care within and between countries during the Covid-19 pandemic: the COVISIT international survey. J Crit Care.

[19] Selman LE, Chao D, Sowden R, Marshall S, Chamberlain C, Koffman J (2020). Bereavement Support on the Frontline of COVID-19: recommendations for Hospital clinicians. J Pain Symptom Manage.

[20] Renckens SC, Pasman HR, Klop HT, du Perron C, van Zuylen L, Steegers MAH (2023). Support for relatives in the intensive care unit: lessons from a cross-sectional multicentre cohort study during the COVID-19 pandemic. BMC Health Serv Res.

[21] Renckens SC, Pasman HR, Jorna Z, Klop HT, Du Perron C, van Zuylen L et al. Varying (preferred) levels of involvement in treatment decision-making in the intensive care unit before and during the COVID-19 pandemic: a mixed-methods study among relatives. BMC Medical Informatics and Decision Making. [in press]. 10.1186/s12911-024-02429-y PMC10863197 38347583

[22] Nederlandse Vereniging voor Intensive Care [Dutch Association for Intensive Care]. Nazorg en revalidatie van intensive care patiënten [Aftercare and rehabilitation of intensive care unit patients]. 2022. Available from: https://richtlijnendatabase.nl/richtlijn/nazorg_en_revalidatie_van_intensive_care_patienten/startpagina_-_pics.html.

[23] Braun V, Clarke V (2006). Using thematic analysis in psychology. Qualitative Res Psychol.

[24] Braun V, Clarke V, Hayfield N, Terry G, Liamputtong P (2019). Thematic Analysis. Handbook of Research Methods in Health Social Sciences.

[25] Saunders B, Sim J, Kingstone T, Baker S, Waterfield J, Bartlam B (2018). Saturation in qualitative research: exploring its conceptualization and operationalization. Qual Quant.

[26] Hennink MM, Kaiser BN, Marconi VC (2017). Code saturation versus meaning saturation: how many interviews are Enough?. Qual Health Res.

[27] Naef R, Peng-Keller S, Rettke H, Rufer M, Petry H (2020). Hospital-based bereavement care provision: a cross-sectional survey with health professionals. Palliat Med.

