# Supplementary material for: Experiences with and needs for aftercare following the death of a loved one in the ICU: a mixed-methods study among bereaved relatives
Source: BMC Palliat Care. 2024 Mar 4;23:65. doi: 10.1186/s12904-024-01396-5 (PMC10910713; doi:10.1186/s12904-024-01396-5)
Supplement: Supplementary file 1 — Supplementary Material 1. [file 12904_2024_1396_MOESM1_ESM.docx]

Appendix 1: Questionnaire

This document shows the questionnaire on support for relatives of ICU patients. There are several versions of the questionnaire (according to time period and whether the patient had died in the ICU) with each mostly containing the same questions. Here we show the questionnaire for relatives of ICU patients who were admitted to the ICU with COVID-19 during the first COVID-19 wave and died.

The questions used in this manuscript are question 50 and 51. These questions were included in all versions of the questionnaire intended for relatives of patients who died in the ICU.

# Questionnaire for relatives of deceased ICU patients with corona

*This questionnaire belongs to the study on support for relatives of ICU patients*

#
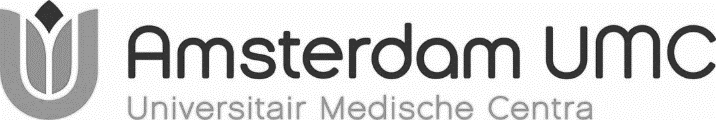


Respondent number

**F 1.2**

# Consent to participate in this study

Before starting this questionnaire, would you please read through the **information letter**? It is enclosed in the same envelope. Then you can tick below whether you want to participate or not.

If you have difficulty understanding the text, you can ask someone close to you or the research team for help at **[email address] or [telephone number].**

Would you like to participate in this study?

⃝ Yes, I want to participate (you can fill in the questionnaire)

⃝ No, I do not want to participate

If you do not wish to participate, you can return the questionnaire in the enclosed reply envelope (no stamp required).

# Instructions

This questionnaire is about your **personal experiences** as a relative. It is only about what you yourself have experienced, heard or witnessed.

Most questions require you to give one answer. If several answers can be given to a question, it says:

*Multiple answers possible*

You make your choice by ticking or colouring the box for the answer of your choice.

Sometimes you will be asked to skip questions. You can then continue at the question indicated:

*Example:* ⃝ No > continue at question ...

You can send the completed questionnaire in the enclosed reply envelope (no stamp required).

# Start of the questionnaire

Has a loved one of yours been admitted to an ICU with corona between mid-March and mid-May 2020?

| ⃝ Yes | ⃝ No, **please note** that this questionnaire is not intended for you. We ask you to return the questionnaire in the enclosed reply envelope (no stamp required). |
| --- | --- |

Were you your loved one's first contact during the time your loved one was in the ICU?

| ⃝ Yes | ⃝ No, **please note** that this questionnaire is not intended for you. We ask you to pass the questionnaire on to the person who was your loved one's first contact person. |
| --- | --- |

#

# Questions about yourself

1. What **are you** of your loved one who was in an ICU between mid-March and mid-May 2020?

| ⃝ Partner/spouse  ⃝ Parent-in-law  ⃝ Other, namely: ________ | ⃝ Parent  ⃝ Brother/sister | ⃝ Child  ⃝ Friend(s) |
| --- | --- | --- |

2. You are…

| ⃝ Man | ⃝ Woman | ⃝ Other |
| --- | --- | --- |

3. How old are you?

| ⃝ Younger than 30 years  ⃝ 66 - 80 years | ⃝ 30 - 50 years  ⃝ Over 80 years old | ⃝ 51 - 65 years old |
| --- | --- | --- |

4. What is your highest completed education (with a degree)?

| ⃝ None  ⃝ Primary education (primary education, preparatory vocational secondary education (vmbo), first three years of senior general secondary education (havo) or university preparatory education (vwo), or vocational training level 1 (mbo)  ⃝ Secondary education (senior general secondary education (havo), university preparatory education (vwo) or vocational training level 2/3/4 (mbo))  ⃝ Higher education (university of applied sciences (HBO), academic university (WO) or PhD) |
| --- |

5. What is your cultural background?

Multiple answers possible

| ⃝ Dutch  ⃝ Turkish | ⃝ Moroccan  ⃝ Antillean | ⃝ Surinamese  ⃝ Other, namely:  ______________________________ |
| --- | --- | --- |

6. Do you have a religion or philosophy?

| ⃝ Yes, namely: ________________________ | ⃝ No |
| --- | --- |

7. In general, how would you describe **your own** health?

| ⃝ Excellent  ⃝ Very good  ⃝ Good  ⃝ Moderate  ⃝ Bad |  |  |
| --- | --- | --- |

8. Have you had corona **yourself**?

| ⃝ Yes, in the same period as my loved one was in the ICU  ⃝ Yes, in a different period from when my loved one was in ICU  ⃝ No |
| --- |

9. How often did you receive support from caregivers **during and shortly after** your loved one's ICU admission?

Please tick the number of times for each caregiver

|  | **0 times** | **1 time** | **2 times** | **3 times** | **≥ 4 times** |
| --- | --- | --- | --- | --- | --- |
| General practitioner | ⃝ | ⃝ | ⃝ | ⃝ | ⃝ |
| Social worker | ⃝ | ⃝ | ⃝ | ⃝ | ⃝ |
| Spiritual counsellor | ⃝ | ⃝ | ⃝ | ⃝ | ⃝ |
| Psychologist | ⃝ | ⃝ | ⃝ | ⃝ | ⃝ |
| Coach | ⃝ | ⃝ | ⃝ | ⃝ | ⃝ |
| General practice-based nurse specialist | ⃝ | ⃝ | ⃝ | ⃝ | ⃝ |
| Victim Support | ⃝ | ⃝ | ⃝ | ⃝ | ⃝ |
| IC helpline IC Connect | ⃝ | ⃝ | ⃝ | ⃝ | ⃝ |
| Self-help group | ⃝ | ⃝ | ⃝ | ⃝ | ⃝ |
| (such as IC Connect's Facebook group or a Facebook group for corona patients) | | | | | |
| Other, namely:  _____________________________ | ⃝ | ⃝ | ⃝ | ⃝ | ⃝ |

# Questions about your loved one who was admitted to the ICU

10. How old was **your loved one** at the time of the ICU admission?

| ⃝ Younger than 30 years  ⃝ 66 - 80 years | ⃝ 30 - 50 years  ⃝ Over 80 years old | ⃝ 51 - 65 years old |
| --- | --- | --- |

11. Did **your loved one** have one or more other diseases at the time of admission?

*Multiple answers possible*

| ⃝ Cancer  ⃝ Cardiovascular disease  ⃝ Lung disease (e.g. COPD or emphysema)  ⃝ Diabetes  ⃝ Dementia | ⃝ No, no other disease  ⃝ Don't know  ⃝ Other disease(s), namely: ____________ _____________________________________ |
| --- | --- |

12. How many days did your loved one spend in the ICU?

| ⃝ Less than 5 days  ⃝ 21 - 30 days | ⃝ 5 - 10 days  ⃝ more than 30 days | ⃝ 11 - 20 days |
| --- | --- | --- |

13. In which month(s) did your loved one stay in the ICU?

Multiple answers possible

| ⃝ March 2020  ⃝ June 2020 | ⃝ April 2020 | ⃝ May 2020 |
| --- | --- | --- |

14. Has your loved one been in an ICU in more than one hospital with corona between mid-March and mid-May 2020?

| Yes, namely on ICUs:  ⃝ in the Netherlands  ⃝ in the Netherlands and abroad  ⃝ Other, namely: _______________________________ | ⃝ No |
| --- | --- |

# Questions about support from the hospital

The following questions are about the support you received from the hospital while your loved one was in the ICU.

*If you want to give further explanations about a particular situation, you can do so at the end of the questionnaire.*

15. Did you receive information on the following topics?

|  | **Yes** | **No** |
| --- | --- | --- |
| About the ICU's operating procedure | ⃝ | ⃝ |
| About ICU visiting arrangements | ⃝ | ⃝ |
| About isolation measures in the ICU | ⃝ | ⃝ |
| About the disease corona | ⃝ | ⃝ |
| About the support that was available for you | ⃝ | ⃝ |

16. Was the information clear to you?

| ⃝ Yes | ⃝ Not completely | ⃝ No | ⃝ Not applicable |
| --- | --- | --- | --- |

17. Was your loved one allowed to receive visitors in the ICU?

Multiple answers possible

| ⃝ Yes, maximum _____ people per day  ⃝ Yes, maximum _____ hours per day  ⃝ Yes, but that changed during the ICU admission, namely: _________________________  ⃝ Yes, other namely: ________________________________________________________  ⃝ No > continue at question 22 |
| --- |

18. Have you visited your loved one in the ICU?

| ⃝ Yes | No, because (multiple answers possible):  ⃝ I was afraid for my own health  ⃝ I had corona myself  ⃝ a housemate had corona  ⃝ the ICU was too far away  ⃝ other, namely: _____________________________ |
| --- | --- |

19. Was it clear to you why fewer or no visitors were allowed in the ICU?

| ⃝ Yes | ⃝ Not completely | ⃝ No | ⃝ Not applicable |
| --- | --- | --- | --- |

20. Was it possible to video call your loved one (e.g. via Skype, Facetime, or alternative)?

| ⃝ Yes, I was only able to see my loved one via video calling (my loved one was not approachable)  ⃝ Yes, I was able to see and speak to my loved one via video calling  ⃝ Yes, but I did not do this  ⃝ No |
| --- |

21. How did you feel about fewer or no visitors being allowed in the ICU?

___________________________________________________________________________

___________________________________________________________________________

22. Were you able to view your loved one's medical records online during the ICU admission?

| ⃝ Yes and I did this  ⃝ Don't know | ⃝ Yes, but I did not do this  ⃝ No |
| --- | --- |

23. From which healthcare provider(s) did you receive information about your loved one's situation?

Multiple answers possible

| *⃝* Nurse(s) who cared for my loved one  ⃝ Other nurse(s) who **did not** care for my loved one  ⃝ Doctor(s) who treated my loved one  ⃝ Other doctor(s) who **did not** treat my loved one  ⃝ Other, namely: __________________________________________________________ |
| --- |

24. With which other hospital healthcare provider(s) did you have contact during your loved one's ICU admission?

Multiple answers possible

| ⃝ (Medical) social worker  ⃝ Spiritual counsellor | ⃝ Psychologist  ⃝ Other, namely: ____________________ |
| --- | --- |

25. Which healthcare provider did you have the most frequent contact with?

Tick one answer

| ⃝ Nurse(s) who cared for my loved one  ⃝ Other nurse(s) who **did not** care for my loved one  ⃝ Doctor(s) who treated my loved one  ⃝ Other doctor(s) who **did not** treat my loved one  ⃝ (Medical) social worker  ⃝ Spiritual counsellor  ⃝ Psychologist  ⃝ Other, namely: __________________________________________________________ |
| --- |

26. How was contact arranged between you and the healthcare provider with whom you had the most frequent contact?

Multiple answers possible

| ⃝ The healthcare provider called me  ⃝ I called the healthcare provider | ⃝ Other, namely: ____________________ _____________________________________ |
| --- | --- |

27. **How often** did you receive information about your loved one's situation?

| ⃝ Once a day  ⃝ Several times a day  ⃝ Less than once a day: ______ times a week  ⃝ Other, namely: __________________________________________________________ |
| --- |

28. Were you satisfied with **how often** you received information about your loved one's situation?

| ⃝ Yes | ⃝ No, preferably more often | ⃝ No, preferably less often |
| --- | --- | --- |

29. Did you receive information about your loved one at a **set time**?

| ⃝ Yes, namely between: ______ and ______ hours | ⃝ No |
| --- | --- |

30. Were you satisfied with **when** you received information about your loved one's situation?

| ⃝ Yes | ⃝ A little | ⃝ No, because:  _________________________ |
| --- | --- | --- |

31. What was discussed in these conversations?

Multiple answers possible

| ⃝ General explanation of corona  ⃝ General explanation of the ICU  ⃝ Changes in my loved one’s medical condition  ⃝ Tests and results  ⃝ Explanation of the treatment my loved one received (e.g. ventilation or medication)  ⃝ Explanation of complications my loved one had (such as delirium or thrombosis)  ⃝ Explanation on stopping treatments  ⃝ Explanation of Post Intensive Care Syndrome (PICS)  ⃝ Explanation of Post Intensive Care Syndrome - Family (PICS-F)  ⃝ My concerns about my loved one in the ICU  ⃝ My concerns about how I and other family were doing  ⃝ My concerns about how I could inform other family and friends about the situation  ⃝ Other, namely: __________________________________________________________ |
| --- |

32. Did healthcare providers give you comprehensible information?

| ⃝ Never | ⃝ Sometimes | ⃝ Often | ⃝ Always | ⃝ n/a |
| --- | --- | --- | --- | --- |

33. Did healthcare providers give you contradictory information?

| ⃝ Never | ⃝ Sometimes | ⃝ Often | ⃝ Always | ⃝ n/a |
| --- | --- | --- | --- | --- |

34. Did healthcare providers take you seriously?

| ⃝ Never | ⃝ Sometimes | ⃝ Often | ⃝ Always | ⃝ n/a |
| --- | --- | --- | --- | --- |

35. Did healthcare providers have enough time for you?

| ⃝ Never | ⃝ Sometimes | ⃝ Often | ⃝ Always | ⃝ n/a |
| --- | --- | --- | --- | --- |

36. Did healthcare providers listen to you carefully?

| ⃝ Never | ⃝ Sometimes | ⃝ Often | ⃝ Always | ⃝ n/a |
| --- | --- | --- | --- | --- |

37. Were you asked to be involved in important treatment decisions?

| ⃝ Never | ⃝ Sometimes | ⃝ Often | ⃝ Always | ⃝ n/a |
| --- | --- | --- | --- | --- |

38. When important decisions were made, was there enough time to discuss your concerns and questions?

| ⃝ Never | ⃝ Sometimes | ⃝ Often | ⃝ Always | ⃝ n/a |
| --- | --- | --- | --- | --- |

39. Did the caregivers offer you emotional support?

| ⃝ Yes | ⃝ A little | ⃝ No |
| --- | --- | --- |

40. What rating from 1 (very poor) to 10 (very good) do you give the healthcare providers from the hospital for supporting you as a loved one?

Rate each type of healthcare provider, or 'not applicable' (N/A)

|  | **1** | **2** | **3** | **4** | **5** | **6** | **7** | **8** | **9** | **10** | **N/A** |
| --- | --- | --- | --- | --- | --- | --- | --- | --- | --- | --- | --- |
| Nurse(s) who cared for your loved one | *⃝* | *⃝* | *⃝* | *⃝* | *⃝* | *⃝* | *⃝* | *⃝* | *⃝* | *⃝* | *⃝* |
| Other nurse(s) **not** caring for your loved one | *⃝* | *⃝* | *⃝* | *⃝* | *⃝* | *⃝* | *⃝* | *⃝* | *⃝* | *⃝* | *⃝* |
| Doctor(s) who treated your loved one | *⃝* | *⃝* | *⃝* | *⃝* | *⃝* | *⃝* | *⃝* | *⃝* | *⃝* | *⃝* | *⃝* |
| Other doctor(s) who **did not** treat your loved one | *⃝* | *⃝* | *⃝* | *⃝* | *⃝* | *⃝* | *⃝* | *⃝* | *⃝* | *⃝* | *⃝* |
| Medical social worker(s) | *⃝* | *⃝* | *⃝* | *⃝* | *⃝* | *⃝* | *⃝* | *⃝* | *⃝* | *⃝* | *⃝* |
| Spiritual counsellor(s) | *⃝* | *⃝* | *⃝* | *⃝* | *⃝* | *⃝* | *⃝* | *⃝* | *⃝* | *⃝* | *⃝* |
| Psychologist | *⃝* | *⃝* | *⃝* | *⃝* | *⃝* | *⃝* | *⃝* | *⃝* | *⃝* | *⃝* | *⃝* |

41. If you could change one thing about the support you received from healthcare providers from the hospital, what would it be?

___________________________________________________________________________

___________________________________________________________________________

___________________________________________________________________________

# Questions about the support during the period that your loved one died

We will ask you some questions about how saying goodbye to your loved one went and how you felt about it. The questions are about the death of your loved one, the eventual farewell and the support from the hospital during this period.

42. Was the death of your loved one unexpected for you?

| ⃝ Yes | ⃝ A little | ⃝ No |
| --- | --- | --- |

43. Were you allowed to say goodbye to your loved one in the ICU?

| ⃝ Yes, with ______ persons | ⃝ No **> continue at question 47** |
| --- | --- |

44. Did you say goodbye to your loved one yourself in the ICU?

| Yes,  ⃝ before the death of my loved one  ⃝ after the death of my loved one | No, because (multiple answers possible):  ⃝ I was afraid for my own health  ⃝ I had corona myself  ⃝ a housemate had corona  ⃝ the ICU was too far away  ⃝ other, namely: _____________________________  **> continue at question 47** |
| --- | --- |

45. Could you say goodbye to your loved one in a private room?

| ⃝ Yes | ⃝ No |
| --- | --- |

46. According to you, which of the following terms fit the way in which your loved one died?

Meerdere antwoorden mogelijk

| ⃝ Quiet  ⃝ Good  ⃝ Sad  ⃝ Hectic  ⃝ Shocking  ⃝ Painful  ⃝ Intimate | ⃝ Inhumane  ⃝ Touching  ⃝ Dignfied  ⃝ Uneasy  ⃝ Peaceful  ⃝ I don’t know |
| --- | --- |

47. What did you **like** about the support that you received in the period that your loved one died?

___________________________________________________________________________

___________________________________________________________________________

48. What did you **dislike** about the support that you received in the period that your loved one died?

___________________________________________________________________________

___________________________________________________________________________

49. What rating from 1 (very poor) to 10 (very good) would you give the support from the hospital in the period that your loved one died?

| ⃝ 1 | ⃝ 2 | ⃝ 3 | ⃝ 4 | ⃝ 5 | ⃝ 6 | ⃝ 7 | ⃝ 8 | ⃝ 9 | ⃝ 10 |
| --- | --- | --- | --- | --- | --- | --- | --- | --- | --- |

50. Did a healthcare professional from the hospital ask you in the weeks/months **after** your loved one died how you were doing?

| ⃝ Yes | ⃝ No |
| --- | --- |

51. Did you have an appointment with an ICU physician after the death of your loved one (e.g., to discuss the ICU admission)?

| ⃝ Yes | No,  ⃝ due to COVID-19 restrictions  ⃝ I did not feel a need  ⃝ the hospital where my loved one was admitted was too far away  ⃝ I did not see/read/hear about this option  ⃝ going back to the hospital could evoke negative emotions  ⃝ other, namely: _______________________________________ |
| --- | --- |

# Questions about how you feel now

Now we will ask you some questions about how you are feeling right now. For each question, tick the box that comes closest to how you have been feeling **over the past week**. Don't think too long about your answer. Your first reaction to each question is probably more reliable than a long-thinking answer.

52. I feel tense or ‘wound up’:

| ⃝ Most of the time | ⃝ A lot of the time | ⃝ From time to time, occasionally | ⃝ Not at all |
| --- | --- | --- | --- |

53. I still enjoy the things I used to enjoy:

| ⃝ Defintely as much  ⃝ Only a little | ⃝ Not quite so much  ⃝ Hardly at all |
| --- | --- |

54. I get a sort of frightened feeling as if something awful is about to happen:

| ⃝ Very definitely and quite badly  ⃝ Yes, but not too badly  ⃝ A little, but it doesn’t worry me  ⃝ Not at all |
| --- |

55. I can laugh and see the funny side of things:

| ⃝ As much as I always could  ⃝ Definitely not so much now | ⃝ Not quite so much now  ⃝ Not at all |
| --- | --- |

56. Worrying thoughts go through my mind

| ⃝ A great deal of the time  ⃝ From time to time but not too often | ⃝ A lot of the time  ⃝ Only occasionally |
| --- | --- |

57. I feel cheerful:

| ⃝ Not at all | ⃝ Not often | ⃝ Sometimes | ⃝ Most of the time |
| --- | --- | --- | --- |

58. I can sit at ease and feel relaxed:

| ⃝ Definitely | ⃝ Usually | ⃝ Not often | ⃝ Not at all |
| --- | --- | --- | --- |

59. I feel as if I am slowed down:

| ⃝ Nearly all the time | ⃝ Very often | ⃝ Sometimes | ⃝ Not at all |
| --- | --- | --- | --- |

60. I get a sort of frightened feeling like ‘butterflies’ in the stomach:

| ⃝ Not at all | ⃝ Occasionally | ⃝ Quite often | ⃝ Very often |
| --- | --- | --- | --- |

61. I have lost interest in my appearance:

| ⃝ Definitely  ⃝ I may not take quite as much care | ⃝ I don’t take as much care as I should  ⃝ I take just as much care as ever |
| --- | --- |

62. I feel restless as I have to be on the move:

| ⃝ Very much indeed | ⃝ Quite a lot | ⃝ Not very much | ⃝ Not at all |
| --- | --- | --- | --- |

63. I look forward with enjoyment to things:

| ⃝ As much as I ever did  ⃝ Definitely less than I used to | ⃝ Rather less than I used to  ⃝ Hardly at all |
| --- | --- |

64. I get sudden feelings of panic:

| ⃝ Very often indeed | ⃝ Quite often | ⃝ Not very often | ⃝ Not at all |
| --- | --- | --- | --- |

65. I can enjoy a good book or radio or television programme:

| ⃝ Often | ⃝ Sometimes | ⃝ Not often | ⃝ Very seldom |
| --- | --- | --- | --- |

Below is a list of things people can sometimes experience after a very stressful or traumatic event. When completing it, think back to your loved one's ICU admission and indicate how much you have been bothered by that problem in **the past month**.

|  | **Not at all** | **A little bit** | **Moderately** | **Quite a bit** | **Extremely** |
| --- | --- | --- | --- | --- | --- |
| 66. Repeated, disturbing, and unwanted memories of the stressful experience? | ⃝ | ⃝ | ⃝ | ⃝ | ⃝ |
| 67. Repeated, disturbing dreams about the stressful experience? | ⃝ | ⃝ | ⃝ | ⃝ | ⃝ |
| 68. Suddenly feeling or acting as if the stressful experience were actually happening again (as if you were actually back there reliving it)? | ⃝ | ⃝ | ⃝ | ⃝ | ⃝ |
| 69. Feeling very upset when something reminded you of the stressful experience? | ⃝ | ⃝ | ⃝ | ⃝ | ⃝ |
| 70. Having strong physical reactions when something reminded you of the stressful experience (for example heart pounding, trouble breathing, sweating)? | ⃝ | ⃝ | ⃝ | ⃝ | ⃝ |
| 71. Avoiding memories, thoughts or feelings related to the stressful experience? | ⃝ | ⃝ | ⃝ | ⃝ | ⃝ |
| 72. Avoiding external reminders of the stressful experience (for example: people, places, conversations, activities, objects or situations)? | ⃝ | ⃝ | ⃝ | ⃝ | ⃝ |
| 73. Trouble remembering important parts of the stressful experience? | ⃝ | ⃝ | ⃝ | ⃝ | ⃝ |
| 74. Having strong negative beliefs about yourself, other people or the world (for example having thoughts such as: I am bad, there is something seriously wrong with me, no one can be trusted, the world is completely dangerous)? | ⃝ | ⃝ | ⃝ | ⃝ | ⃝ |
| 75. Blame yourself or someone else for the stressful experience or what happened after it? | ⃝ | ⃝ | ⃝ | ⃝ | ⃝ |
| 76. Having strong negative feelings such as fear, horror, anger, guilt or shame? | ⃝ | ⃝ | ⃝ | ⃝ | ⃝ |
| 77. Loss of interest in activities that you used to enjoy? | ⃝ | ⃝ | ⃝ | ⃝ | ⃝ |
| 78. Feeling distant or cut off from other people? | ⃝ | ⃝ | ⃝ | ⃝ | ⃝ |
| 79. Trouble experiencing positive feelings (for example being unable to feel happiness or have loving feelings for people close to you)? | ⃝ | ⃝ | ⃝ | ⃝ | ⃝ |
| 80. Irritable behaviour, angry outbursts or acting aggressively? | ⃝ | ⃝ | ⃝ | ⃝ | ⃝ |
| 81. Taking too many risks or doing things that could cause you harm? | ⃝ | ⃝ | ⃝ | ⃝ | ⃝ |
| 82. Being "superalert” or watchful or on guard? | ⃝ | ⃝ | ⃝ | ⃝ | ⃝ |
| 83. Feeling jumpy or easily startled? | ⃝ | ⃝ | ⃝ | ⃝ | ⃝ |
| 84. Having difficulty concentrating? | ⃝ | ⃝ | ⃝ | ⃝ | ⃝ |
| 85. Trouble falling asleep or staying asleep? | ⃝ | ⃝ | ⃝ | ⃝ | ⃝ |

Finally, below several grief reactions are listed. Tick one of the boxes to the right of the description to indicate the number of times you have had these reactions **in the** **past month**, in response to the the death of your loved one.

|  | **Never** | **Rarely** | **Sometimes** | **Frequently** | **Always** |
| --- | --- | --- | --- | --- | --- |
| 86. I had intrusive thoughts or images related to the person who died. | ⃝ | ⃝ | ⃝ | ⃝ | ⃝ |
| 87. I experienced intense emotional pain, sadness, or pangs of grief. | ⃝ | ⃝ | ⃝ | ⃝ | ⃝ |
| 88. I found myself longing or yearning for the person who died. | ⃝ | ⃝ | ⃝ | ⃝ | ⃝ |
| 89. I experienced confusion about my role in life or a diminished sense of self. | ⃝ | ⃝ | ⃝ | ⃝ | ⃝ |
| 90. I had trouble accepting the loss. | ⃝ | ⃝ | ⃝ | ⃝ | ⃝ |
| 91. I avoided places, objects, or thoughts that reminded me that the person I lost had died. | ⃝ | ⃝ | ⃝ | ⃝ | ⃝ |
| 92. It was hard for me to trust others. | ⃝ | ⃝ | ⃝ | ⃝ | ⃝ |
| 93. I felt bitterness or anger related to his/her death. | ⃝ | ⃝ | ⃝ | ⃝ | ⃝ |
| 94. I felt that moving on (e.g. making new friends, pursuing new interests) was difficult for me. | ⃝ | ⃝ | ⃝ | ⃝ | ⃝ |
| 95. I felt emotionally numb. | ⃝ | ⃝ | ⃝ | ⃝ | ⃝ |
| 96. I felt that life is unfulfilling or meaningless without him/her. | ⃝ | ⃝ | ⃝ | ⃝ | ⃝ |
| 97. I felt,stunned, shocked or dazed by his/her death. | ⃝ | ⃝ | ⃝ | ⃝ | ⃝ |
| 98. I noticed significant reduction in social, occupational, or other important areas of functioning (e.g. domestic responsibilities) as a result of his/her death. | ⃝ | ⃝ | ⃝ | ⃝ | ⃝ |
|  | **Never** | **Rarely** | **Sometimes** | **Frequently** | **Always** |
| 99. I had intrusive thoughts and images associated with the circumstances of his/her death. | ⃝ | ⃝ | ⃝ | ⃝ | ⃝ |
| 100. I experienced difficulty with positive reminiscing about the lost person. | ⃝ | ⃝ | ⃝ | ⃝ | ⃝ |
| 101. I had negative thoughts about myself in relation to the loss (e.g., thoughts about self-blame) . | ⃝ | ⃝ | ⃝ | ⃝ | ⃝ |
| 102. I had a desire to die in order to be with the deceased. | ⃝ | ⃝ | ⃝ | ⃝ | ⃝ |
| 103. I felt alone or detached from other individuals. | ⃝ | ⃝ | ⃝ | ⃝ | ⃝ |

# In conclusion

We would like to get in touch with you to ask additional questions about your experiences. We would like to do this through a one-off **telephone interview** lasting about 30 minutes. You can always decide not to participate at a later date.

May we contact you for a telephone interview?

| ⃝ Yes, you may contact me to ask if I would like to participate in an interview.  My phone number: __________________________________  My email address: _______________________________________ | ⃝ No |
| --- | --- |

If you would like to say anything else about the questionnaire, the survey or if you have any other comments, please write it down below:

___________________________________________________________________________

___________________________________________________________________________

___________________________________________________________________________

# Thank you very much for completing this questionnaire!

You can send the completed questionnaire in the **enclosed reply envelope** (no stamp required). If you have any further questions, please contact us using the details below.

It could be that memories of the period you went through are upsetting you. If you want to talk to a healthcare provider about this, please contact the independent doctor [name], she can refer you to the right person.

| **Executive researcher:**  Sophie Renckens  [telephone number]  [email address] | **Principal investigator:**  Prof. Dr Bregje Onwuteaka-Philipsen  [email address] |
| --- | --- |


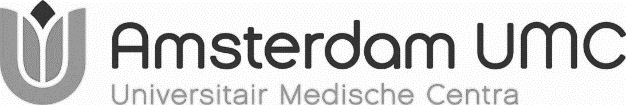
**Independent doctor:**

[Name]

[email address]

[telephone number]
