# Supplementary material for: Experiences with and needs for aftercare following the death of a loved one in the ICU: a mixed-methods study among bereaved relatives
Source: BMC Palliat Care. 2024 Mar 4;23:65. doi: 10.1186/s12904-024-01396-5 (PMC10910713; doi:10.1186/s12904-024-01396-5)
Supplement: Supplementary file 2 — Supplementary Material 2. [file 12904_2024_1396_MOESM2_ESM.docx]

Appendix 2: Topic list interview

- Global description of the ICU admission
- Experiences with the visitation policy
- Experiences with support during the ICU admission
  - Global description of the received support from the hospital
    - Additional support outside the hospital (e.g. general practitioner)
  - Satisfaction with support and important elements
  - Support around the end-of-life (if patient deceased in the ICU)
- Experiences with treatment decision-making during the ICU admission
  - Global description of treatment decision-making
  - Satisfaction with involvement in treatment decision-making
  - Wishes and needs regarding involvement in treatment decision-making
- Experiences with aftercare for relatives (both when a patient was discharged and when a patient deceased in the ICU)
  - Global description of the received aftercare
  - Satisfaction with the aftercare
  - Wishes and needs regarding aftercare
